# Supplementary material for: The representation of omitted sounds in the mouse auditory cortex
Source: Nat Commun. 2026 Jan 28;17:2107. doi: 10.1038/s41467-026-68847-w (PMC12953763; doi:10.1038/s41467-026-68847-w)
Supplement: Supplementary file 1 — Supplementary Information [file 41467_2026_68847_MOESM1_ESM.pdf]

Supplementary Figures

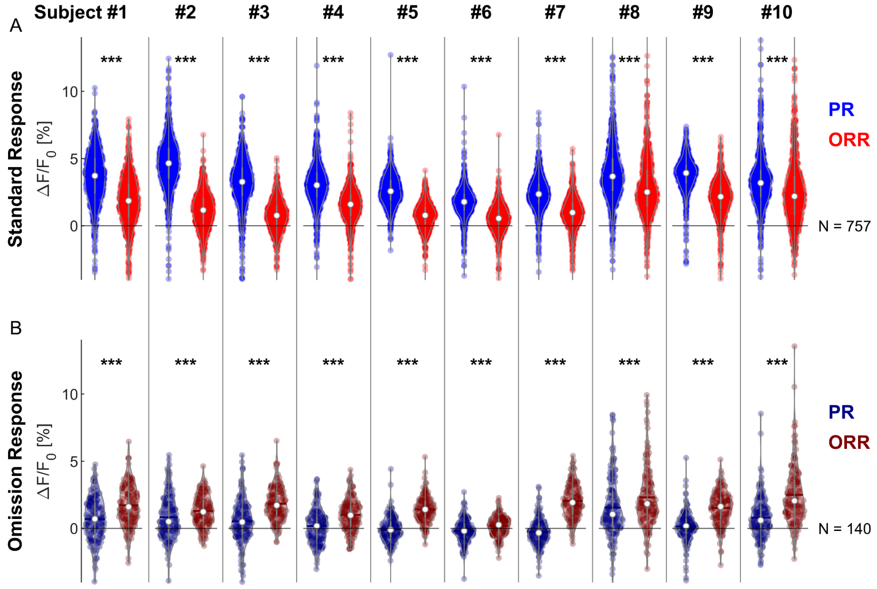

Supp. Figure 1: The Omission region responds more strongly to omissions, while the Primary region responds more strongly to stimuli

A For all subjects (N=10 mice), the PR showed a stronger response to the stimulus than the ORR across trials ($p<0.001$, Wilcoxon ranksum test).

B For all subjects (N=10 mice), the ORR showed a stronger OR than the PR across trials ($p<0.001$, Wilcoxon ranksum test).

We excluded all stimuli/omissions which were preceded by less than 3 standards. Source data are provided with this paper.

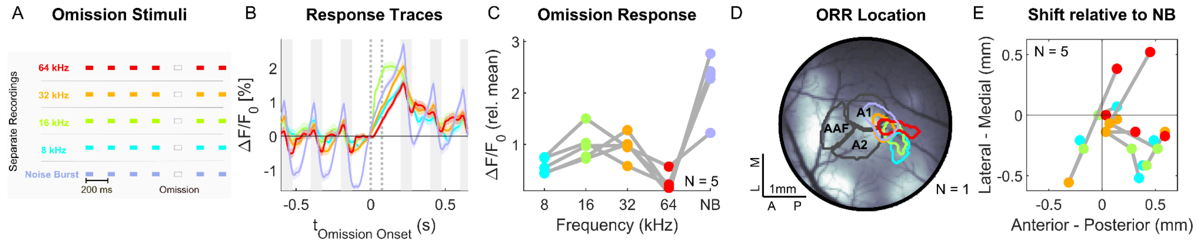

Supp. Figure 2: Omission responses for different stimuli

A The 200 SOA omission paradigm was run in different experiments with one of 5 different stimuli, 4 pure tones [8,16,32,64] kHz, or a broadband noise burst [2-64 kHz]. All stimuli were 75 ms in length and presented at 50 dB SPL within the same session, and all sequence properties were identical to the standard 200 SOA sequence. Colors indicate the type of stimulus.

B In the ORR - defined by the omission response per paradigm - the size of the omission response (OR) showed some variability, as did the stimulus response.

C The OR across the different stimuli showed variability with a tendency towards highest ORs for Noise bursts, and lowest responses to the 64 kHz sound (dF values per animal normalized by within-animal mean, N=5 mice)

D The ORRs for the different stimuli were located in similar areas for a representative animal.

E Across animals (N=5 mice), the localization of the center of the ORR showed no clear spatial gradient or relative pattern for different stimulus frequencies, though centers of Pure Tone ORRs tended to be shifted Posterolateral with respect to the center of Noise Burst ORRs (located at [0,0] for each animal). Source data are provided with this paper.

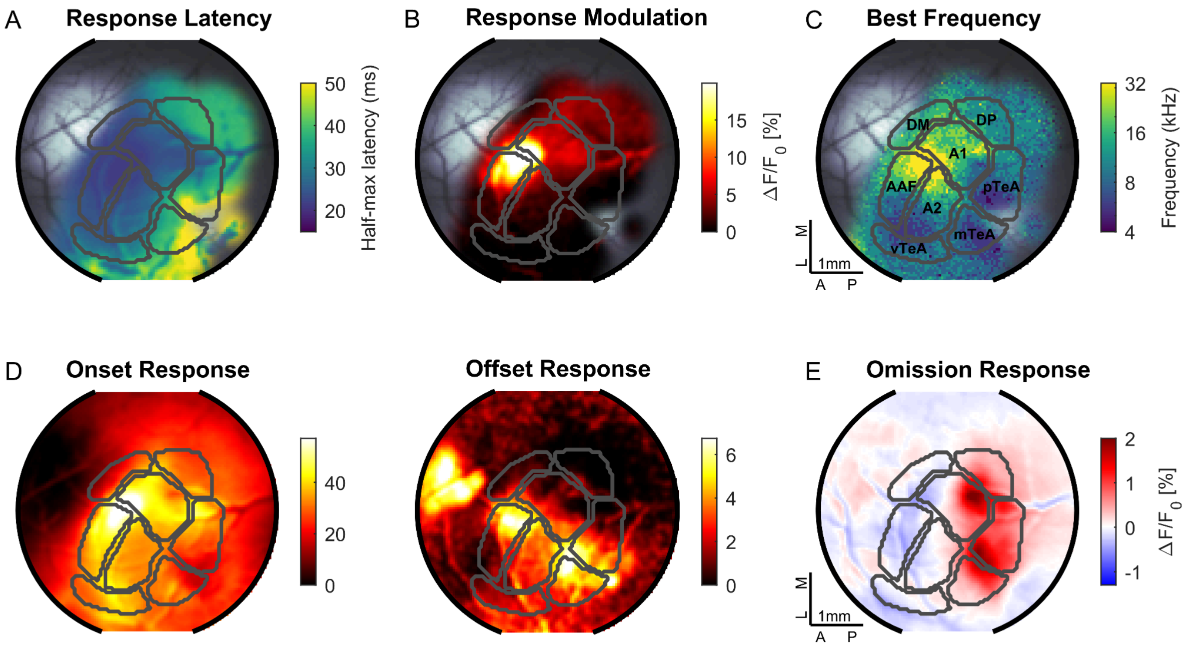

Supp. Figure 3: Fast and clear jGCaMP8m response enables area-division of auditory cortex.

A The fast rise-time of the indicator allowed clear localization of a low-latency core of the auditory cortex by interpolating the latency at which activity reached half of the local maximum. We interpreted these core regions to encompass the primary regions A1, A2 and AAF and based the subdivision on the tonotopic axis (shown in C).

B Similarly, the degree of activity modulation within the 75 ms stimulus window showed a clear, strong-modulation core. Colormap transparency in A, B & C indicates the degree to which the response is above noise floor (i.e. metric derived by combining maps in D&E, see Methods for details).

C Best-Frequency estimate based on responses to Pure Tone stimuli of varying Frequency and Amplitude. Areas around the core regions were considered to be secondary regions and manually divided based on the tonotopy and in accordance with divisions from the Allen Brain Atlas as well as previous literature (see Methods for details).

D Onset responses were defined as the peak activity value that occurred before and after stimulus offset, respectively. To minimize the confound of response summation, offset response amplitude was measured relative to the activity level before stimulus offset. While A1 and A2 showed the strongest onset responses, offset responses were smaller and more homogeneously spread over primary and secondary areas with vTeA showing the most salient difference between onset and offset response.

E Omission responses, in contrast to both onset and offset responses, were on average strongest in the medial and posterior Temporal association area, indicating that they are driven by separate neural subpopulations. In contrast to Fig. 1G, here the omitted stimulus was Noise Burst, which produces wider and stronger omission responses (see also Fig. S2).

Data shown in this figure come from a single representative animal, same as in Figure 1. Source data are provided with this paper.

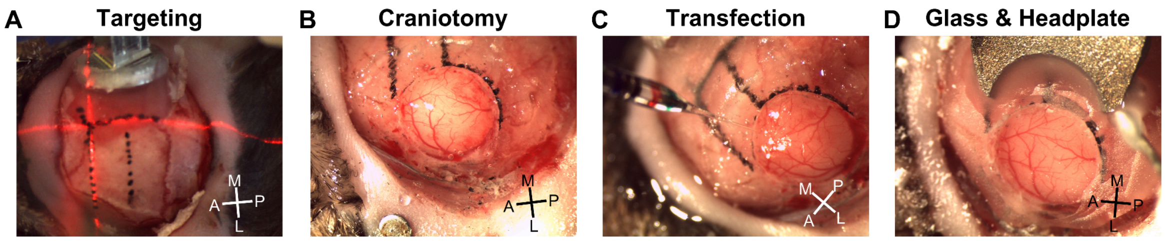

Supp. Figure 4: Surgical Procedure for viral transfection and chronic optical imaging.

A A cross-laser mounted orthogonally to the top of the skull was used in combination with a surgical caliper to determine the center of the craniotomy based on the point-to-point distance to Bregma (laser cross in the image is aligned with bregma, parallel dotted line indicates 2.4mm posterior).

B The curved skull was leveled with transparent dental cement to serve as a flat surface surrounding the craniotomy. A craniotomy was performed under sterile conditions using a combination of pneumatic drill (0.05 mm burr size) and a #4 forceps, to expose the dura mater.

C Keeping the dura moist with sterile saline, viral solution was slowly injected in multiple evenly spaced locations, using a pulled glass capillary, angled at ~45° with respect to the brain surface.

D A sterilized stack of thin glass coverslips was placed carefully on the exposed tissue and cemented in place, preventing the brain from bulging out and allowing for a clear view of the transfected brain areas. Lastly, a flat titanium headplate was mounted on the skull in order to allow for pain-free and accurate head fixation of the awake animal. Source data are provided with this paper.

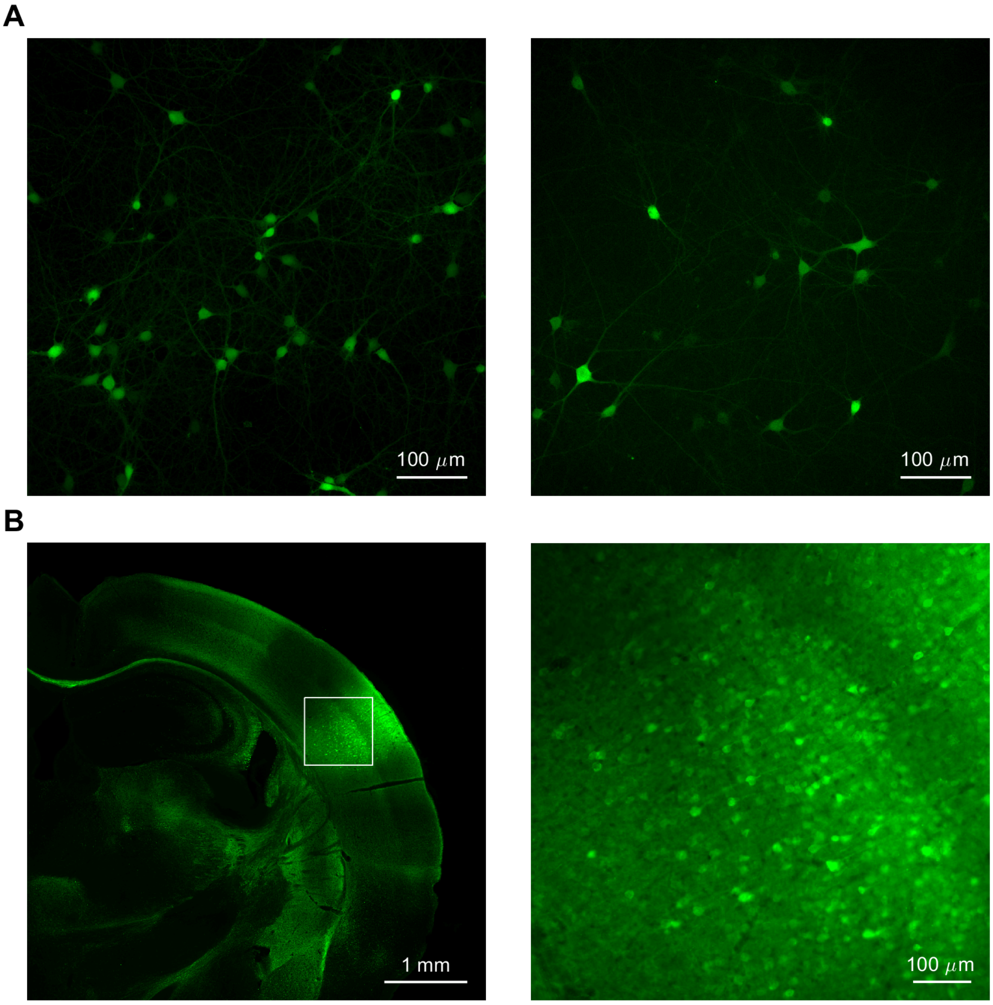

Supp. Figure 5: Histological verification of expression of Ca^{2+} reporter jGCaMP8m.

A To select between serotype 1 or 9 of AAV (adeno-associated virus), we expressed jGCaMP8m in rat hippocampal culture neurons. Expression level and quality was comparable between AAV1 (left) and AAV9 (right, 10 days *in vitro*, same illumination intensity). AAV9 was selected for *in vivo* expression.

B After transfection with AAV9-jGCaMP8m (50 nl, 1×10^{13} vg/ml, 200 μm injection depth) in the left auditory cortex *in vivo*, expression was verified in coronal slices (3 mm post-bregma), showing clear expression in the auditory cortex (left: hemisphere, right: zoom to auditory cortex). Source data are provided with this paper.

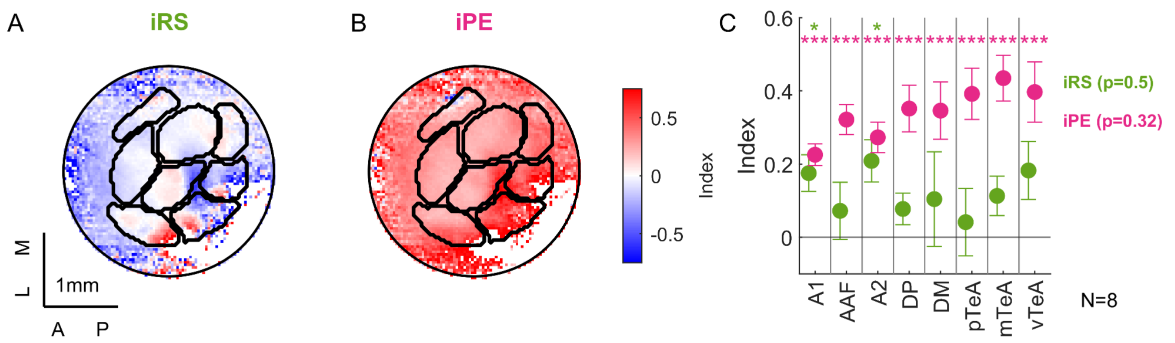

Supp. Figure 6: Indices of repetition suppression and Prediction error

A & B In Fig. 7 repetition suppression (RS) and prediction errors (PE) were evaluated in terms of the actual response strength. Previous work in the literature has alternatively defined indices, which quantify the same measures relative to each pixel's response strength to the control stimulus (Parras et al. 2017). These are referred to as the index of repetition suppression (iRS) and index of prediction error (iPE), calculated as (MSC-STD)/(MSC+STD) & (DEV-MSC)/(DEV+MSC). While these indices have the advantage of locally evaluating the relative size of the different responses, they can also emphasize small responses, which is visible here in the areas surrounding the auditory cortex (compare to Fig. 7). To prevent strong overemphasis, pixels with responses <0.001% (dF/F) above the out-of-craniotomy mean in any of the conditions were excluded from analysis (set to 0).

C Across animals (N=8 mice) we find that iPE was significantly greater than 0 in all areas, with a non-significant tendency towards higher values in the secondary areas p/m/vTeA, which was quite different from the PE values (Fig. 7). Conversely iRS showed a similar tendency to RS with only significant values in A1 and A2, while the difference across areas was not significant in this case. While we think this comparison of different indices is important in relation to previous studies, it also highlights that the choice of measure can influence the results. Significance was assessed using Kruskal-Wallis ANOVA for iRS and iPE across areas (p-values on the right), and a significance star per area indicates p<0.05 tested by Wilcoxon signrank test including false discovery rate correction via Benjamini-Hochberg. Source data are provided with this paper.

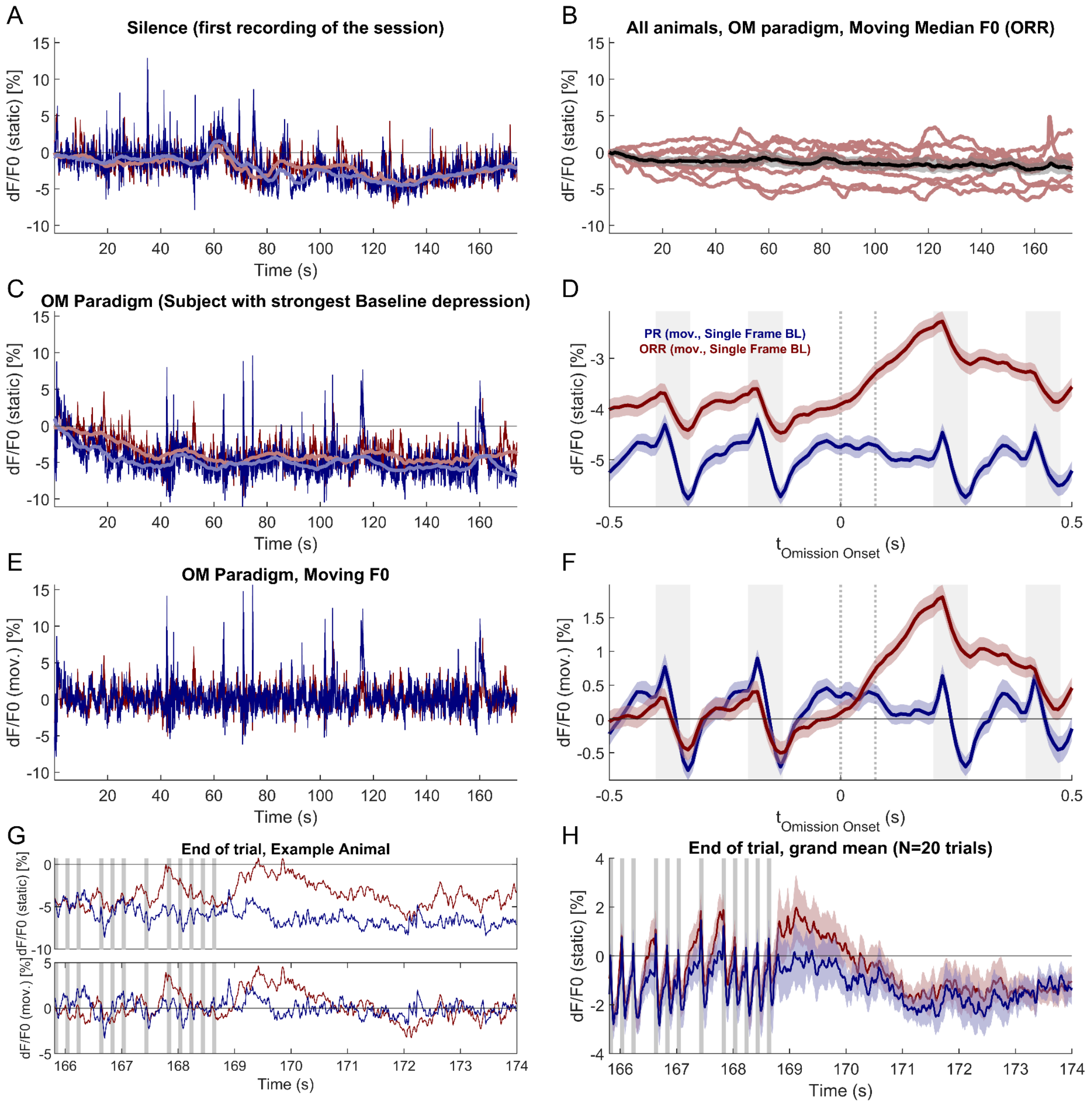

Supp. Figure 7: Moving-median baselining effectively combats within-trial baseline drift while retaining trace shape characteristics.

All traces in the figure are derived from the PRs (blue)/ORRs (red) for the SOA 200 omission paradigm.

A Baseline variations in silence are of the same order as those under acoustic stimulation (**C**). Data here shown with static baseline, i.e. F_0 was set to the average of the first 5s (before stimulus onset). Moving median baseline (8s window) overlaid as thick line, in corresponding, brighter color.

B Across animals, the baseline variation under the omissions paradigm traces show different trends, with some traces increasing and some decreasing over time. Overall, only a small reduction in fluorescence is observed (black, gray indicates SEM).

C Next, we show the effect of moving baselining on the animal that showed the strongest depression, here shown with static baseline (i.e. same principle as for silent recording in **A**). Note that the negative transient at the beginning happens before stimulus presentation and appears to be random. For a detailed view of the post-stimulus response see **G**).

D Without dynamic baselining, the ORR and PR traces are entirely negative and offset from each other, but the omission response is only visible in the ORR, suggesting that the omission response is not caused by a global return to baseline F0 during omissions, in particular as the more negative

baseline in PR would predict a stronger return to baseline, which is not observed (Errorbars indicate SEM over N=200 omissions, same in **F**)
E Same data as in **C/D** with moving median baseline (8s window), globally detrends the traces, leaving local dynamics intact.
F With dynamic baselining, the traces of both regions are centered on 0, while the local shape of the traces remains largely unaffected (compared to **D**).
G In the example animal, offset of the stimuli (gray) causes transient activation particularly in the ORR, after which the trace settles back towards peri-stimulation levels, consistent with the present moving baseline estimate (compare top (static baseline) to bottom (moving baseline)).
H Across all animals and trials, this trend is confirmed, here shown in the static baseline: After the end of the last stimulus, the traces settle back to the moving baseline, rather than returning to 0. In the ORR, the omission response is clearly visible, showing multiple peaks which show similar spacing as the stimulus sequence suggesting some degree of entrainment (however, see response to R3 on entrainment). Note that we only have 2 trials per animal, i.e. 20 trials in total collected for all animals (Errorbar: SEM over N=10 mice), for the present analysis, which leads to the poorer SNR here in comparison to the average responses within the sequence. Source data are provided with this paper.

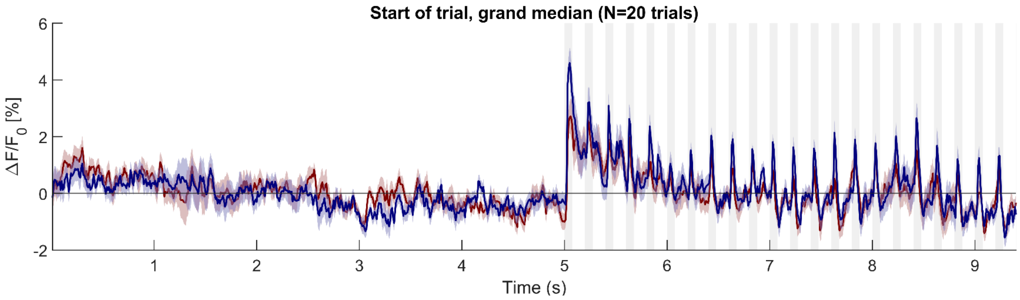

Supp. Figure 8: Stimulus specific adaptation and gradual response shape morphing occurs after repeated stimulation at the beginning of the sequence.

Data in this plot is derived from the median over both trials of all 10 mice for the SOA 200 paradigm, for ORR (red) and PR (blue), aligned to recording start. A large and relatively undefined response occurs for the first stimulus in the PR, accompanied by a slower excitatory modulation (~0.5s half-decay time) across both areas. With an increasing number of stimuli, a fast baseline undershoot develops after the stimulus onset response, sharpening the onset response (see Fig. 1G for mean response shape after sequence habituation has occurred, Errorbar: SEM over N=10 mice). Source data are provided with this paper.

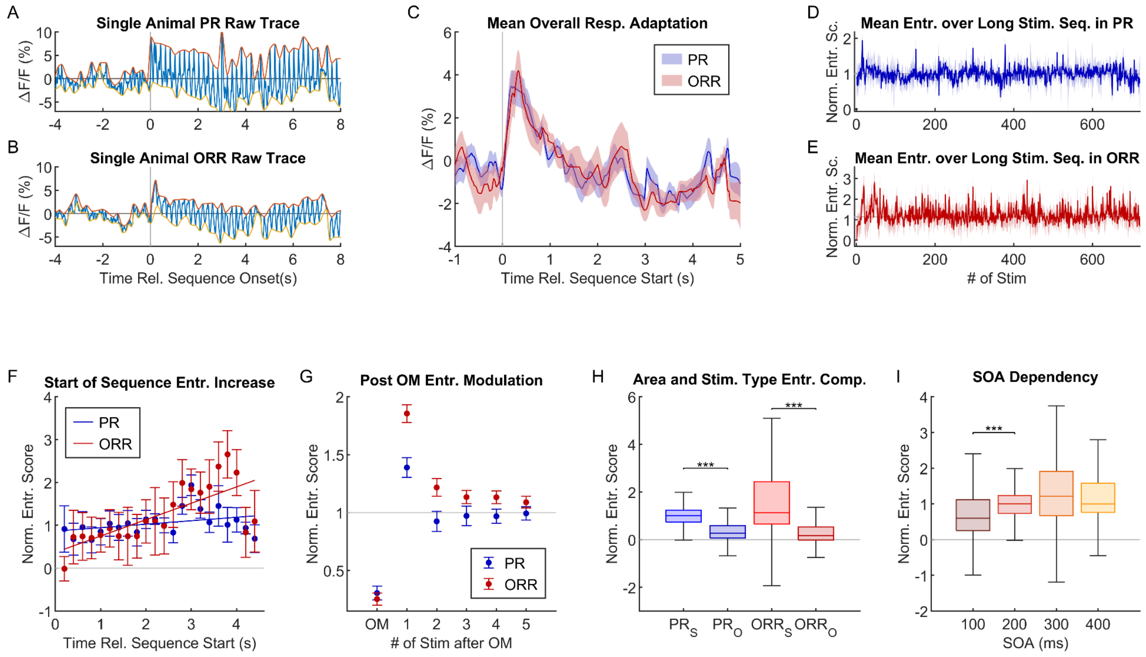

Supp. Figure 9: Sound responses, but not omissions, are subject to entrainment across areas. Entrainment score for each stimulus was defined as the amplitude difference between the response peak and trough within an empirically selected window starting at stimulus onset and going to 50 ms after stimulus offset (for 100 ms SOA, only 25 ms), measuring the similarity to the average stimulus response shape, after normalization (see below).

A & B Example single-animal, raw ΔF/F traces aligned to sequence onset for PR (A) and ORR (B) respectively. Envelopes were drawn with interpolation between peaks (orange) and troughs (yellow) as a visual indicator of entrainment score.

C The mean overall response was calculated by first averaging the upper and lower envelopes per animal, and then computing the mean across animals. For both areas, the overall response size rapidly adapted over the course of ~10 stimuli (2s).

D & E Mean, normalized entrainment score across all animals over an entire trial for PR (D) and ORR (E) respectively. Following an early, fast rise and slower decline, scores stabilized with fluctuations around a steady level. Normalized entrainment score for each animal was obtained by dividing its raw entrainment score by the median raw entrainment score of the primary region across all stimuli.

F The initial entrainment increase at the start of the stimulus sequence. Solid lines indicate linear fits of entrainment scores. ORR exhibited a faster and more pronounced increase than PR.

G Mean entrainment scores were computed for omissions and the stimuli following them, averaged across all omission events. Entrainment scores were significantly lower during omissions periods compared with stimulus periods. The first stimulus after an omission exhibited significantly higher entrainment than baseline, followed by a rapid return to baseline levels.

H Distribution of entrainment scores during stimulus and omission periods was assessed across both areas and all animals. In both areas, entrainment scores during stimuli were significantly higher than during omissions ($p<10^{-10}$, linear mixed-effects model, group effect, animal as random factor), whereas asymptotic entrainment values did not differ significantly between areas.

I Entrainment scores were computed across paradigms with different SOAs. Scores increased significantly from 100 to 200 ms SOA ($p<10^{-10}$, as above), and remained stable between 200 and 400 ms SOA. This pattern is partly consistent with Fig. 5Q.

The quartiles and errorbars in panels C-I indicate SEM across N=7 mice. Source data are provided with this paper.

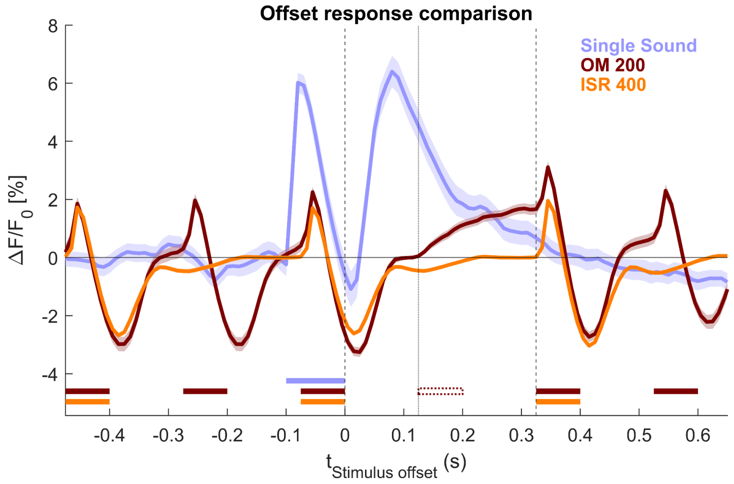

Supp. Figure 10: Comparison of offset with omission responses.

Traces were temporally aligned to stimulus offsets for a single, 100 ms sound (light blue, same Paradigm as Fig. 1C, 40 trial average) and the OM200 (maroon, 200 trials = omissions) / ISR400 (orange, 1440 trials = sounds) responses in the ORR of the same example animal. Within-sequence traces were baselined using 8s moving median over the entire sequence, the Noise burst trace was baselined to the pre-stimulus period (400 ms window). Stimuli are indicated as horizontal bars, and between the vertical dashed lines, there was no stimulus present in all conditions. The omission in the OM200 condition is indicated as an empty, dashed rectangle. Isolated sounds are followed by a fast offset response, whose initial dynamics can also be seen in the sequence conditions. During the offset response's decay period, the within-sequence data shows no decrease, and the OM200 shows the OR's characteristic ramping, inflecting at omission onset (dotted vertical line), which is absent in the ISR400. Data shown in this figure come from a single representative animal (same as in Fig. 7). Errorbars indicate SEM based on the number of trials indicated above for the different conditions. Source data are provided with this paper.
