# Supplementary material for: The representation of omitted sounds in the mouse auditory cortex
Source: Nat Commun. 2026 Jan 28;17:2107. doi: 10.1038/s41467-026-68847-w (PMC12953763; doi:10.1038/s41467-026-68847-w)
Supplement: Supplementary file 2 — Description of Additional Supplementary Files [file 41467_2026_68847_MOESM2_ESM.pdf]

Description of Additional Supplementary Files

Supplementary Data 1: Collection of detailed statistics for all comparisons
